# Major range extensions for two genera of the parasitoid subtribe Facitorina, with a new generic synonymy (Braconidae, Rogadinae, Yeliconini)

**DOI:** 10.3897/zookeys.584.7815

**Published:** 2016-04-26

**Authors:** Buntika A. Butcher, Donald L. J. Quicke, Santhosh Shreevihar, Avunjikkattu Parambil Ranjith

**Affiliations:** 1 Department of Biology, Faculty of Science, Chulalongkorn University, Phayathai Road, Pathumwan, BKK 10330, Thailand; 2 Insect Ecology and Ethology Laboratory, Department of Zoology, University of Calicut, Kerala, Pin: 673635, India; 3 Department of Zoology, Malabar Christian College, Kozhikode (Affiliated to University of Calicut), 673001, Kerala, India

**Keywords:** New distribution record, new species, new synonymy, parasitoid

## Abstract

The genera *Conobregma* van Achterberg and *Facitorus* van Achterberg are recorded from the Afrotropical region and the Indian subcontinent, respectively, for the first time, and two new species are described and illustrated: *Conobregma bradpitti* Quicke & Butcher, **sp. n.** from South Africa and *Facitorus nasseri* Ranjith & Quicke, **sp. n.** from India. *Conobregma bradpitti* **sp. n.** is intermediate between *Conobregma* which was described originally from the New World, and *Asiabregma* Belokobylskij, Zaldivar-Riverón & Maetô, which was coined for the S. E. Asian and East Palaearctic (Japanese) species described under the name *Conobregma*, plus more recently discovered taxa, but the differences between these genera are few and slight. Of the four previously proposed diagnostic characters for separating *Asiabregma* from *Conobregma*, the new species shares two with each, and therefore, the two genera are formally synonymised. *Facitorus* was previously known only from the East Palaearctic region and from S. E. Asia (Japan, Nepal, Taiwan and Vietnam).

## Introduction

The Facitorini were originally described as a tribe in the subfamily Betylobraconinae Tobias, 1979 based on the genera *Facitorus* van Achterberg from Nepal, China and Taiwan, *Conobregma* van Achterberg from the USA and Dominican Republic, and *Jannya* van Achterberg from Colombia and Costa Rica (van Achterberg 1995a). Despite all taxa placed in the Betylobraconinae being morphologically highly derived with robust femora, and shortened tarsi, moderately to very bulging faces, and curved fore wing vein M+CU (van Achterberg 1995a) they have subsequently been shown not to be monophyletic (Zaldivar-Riverón et al. 2006). The morphological homoplasy of these characters even led van Achterberg (1991) to place the rogadine tribe Yeliconini in the Betylobraconinae, though this arrangement was soon dropped as a result of further consideration of biological and morphological evidence. The Facitorini were transferred to the subfamily Rogadinae as a subtribe of the Yeliconini by Belokobylskij et al. (2008) on the basis of DNA sequence data and this placement has been supported by subsequent studies (Zaldivar-Riverón et al. 2009, Butcher et al. 2014, Quicke and Butcher 2015). Most recently, Butcher and Quicke (2015) formally synonymised Betylobraconinae with the Rogadinae maintaining it as a separate tribe. Unfortunately, nothing is yet known about the biology of the Facitorina though their similarity to *Yelicones*, which is a koinobiont larval endoparasitoid of Lepidoptera larvae concealed to some extent in silk webs, suggests that they may have similar biology.

Shortly after the original description of *Conobregma*, which was based on New World species, van Achterberg (1995b) added a new species from the Indonesian island of Sulawesi, thus extending the apparent distribution of the genus to the Old World tropics. Discovery of additional specimens of another genus, *Aulosaphobracon* Belokobylskij & Long, as well as DNA sequence data led Belokobylskij et al. (2008) to coin a new genus, *Asiabregma*, for the Asian and East Palaearctic species that fell within van Achterberg’s concept of *Conobregma*. However, despite their very disjunct distribution, the two genera were only separated by four, rather weak, characters (Table 1), and the new species from S. Africa is rather intermediate. We therefore synonymise *Asiabregma* with *Conobregma*, and treat the new species under the latter name.

**Table 1.** Differences used by Belokobylskij et al. (2008) to differentiate between *Conobregma* and *Asiabregma*.

Characters	*Conobregma*	*Conobregma bradpitti* sp. n.	‘*Asiabregma*’
Claw of middle leg	short, not pectinate	long and pectinate	long and pectinate
Postpectal carina	absent	absent	distinct
Fore wing vein 2CUa	short <= m-cu	short = m-cu	long > twice m-cu
Carina between eye and antennal sockets	absent	present	present

## Materials and methods

The holotype of *Conobregma bradpitti* sp. n. is deposited in the Hymenoptera Institute Collection, Department of Entomology, University of Kentucky, Lexington, Kentucky. It was imaged using an Olympus SXZ16 microscope with automated multiple image capture at preset focal levels using an Olympus DP72 camera, and image combination using the Cell^D image processing system. The specimen was card-mounted and rather fragile but we successfully remounted it to enable more features to be seen.

The holotype of *Facitorus nasseri* sp. n. is deposited in the Department of Zoology, University of Calicut, Kerala, India. It was imaged using an Leica M205A stereomicroscope with automated multiple image capture at preset focal levels using an Leica DMC 2900 camera, and image combination using the Leica Application Suite image processing system v4.7. All images were edited using Photoshop CS6 (Version 6.1) (Adobe Inc.).

Terminology follows van Achterberg (1988) except for wing venation nomenclature which follows Sharkey and Wharton (1997); see also Figure 2.2 in Quicke (2015) for comparison of wing venation naming systems.

## Descriptive taxonomy

### Conobregma bradpitti

Taxon classificationAnimaliaHymenopteraBraconidae

Quicke & Butcher sp. n.

http://zoobank.org/4C0937AE-13E0-43F2-B411-1CFFCB881FD6

Figures 1–4 , 5–6

#### Material examined.

Holotype female: “South Africa, Madlangula, Kosi Bay, 14.iii – 30.iv.1985, R. Kyle”.

#### Diagnosis.

*Conobregma bradpitti* sp. n. may be distinguished from the East Palaearctic and East Asian species (*Conobregma makiharae* (Belokobylskij, Zaldivar & Maetô, 2008), *Conobregma ryukyuensis* (Belokobylskij, Zaldivar & Maetô, 2008)) and *Conobregma sulaensis* van Achterberg, 1995) by fore wing vein 2CUa being approximately the same length as m-cu rather than approximately twice as long. It may be distinguished from all the New World species except for *Conobregma cometes* van Achterberg, 1995 by having the third metasomal tergite almost entirely smooth. It differs from *Conobregma cometes* in having the mesoscutum coarsely sculptured with deep depressions at the bases of setae rather than being coriaceous, and by having the propodeum distinctly less strongly sculptured antero-laterally.

#### Description (female).

Length of body 1.75 mm, and of fore wing 1.5 mm.

Head. Antennae broken. First flagellomere 1.05 × longer than 2^nd^ and 3^rd^ respectively; approximately 1.8 × longer than apically wide, expanding from base to apex. Width of head: width of face: height of eye = 1.0: 0.5: 0.42. Eyes glabrous, with distinct curving fine ridge between antennal socket and eye. Distance between posterior ocelli: transverse diameter of posterior ocellus: shortest distance between posterior ocellus and eye = 1.0: 1.0: 2.5. Frons and occiput smooth. Occipital carina complete.

Mesosoma. Mesosoma 1.8 × longer than high. Propleuron largely smooth. Mesoscutum irregularly sculptured, with deep pits at bases of setae, these forming very conspicuous submarginal rows; with rugulose sculpture between notauli posteriorly. Notauli deeply impressed and strongly sculptured. Precoxal sulcus running from anterior margin to just posterior of mid-length of metapleuron, rugulose. Mesopleuron and mesosternum otherwise largely smooth. Median area of metanotum with weak mid-longitudinal ridge. Propodeum largely foveate except for pair of triangular areas anteriorly on either side of mid-line which are finely aciculate; with short mid-longitudinal carina anteriorly.

Wings. Pterostigma 2.1 × longer than its maximum width. Fore wing vein r-rs approximately 0.65 × maximum width of pterostigma. Lengths of fore wing veins r-rs: 3RSa: 3RSb = 1.0: 3.0: 5.5. Lengths of fore wing veins CU1a: CU1b = 1.0:1.25.

Legs. Fore femur: tibia: tarsus = 1.3: 1.25: 1.0. Fore basitarsus 1.5 × longer than next three articles combined. Mid-tibial claw with well-developed, pecten. Hind femur: tibia: tarsus = 1.0: 1.2: 1.2.

Metasoma. Second metasomal tergite with fine longitudinal striation and interconnecting transverse ridges; approximately 1.8 × longer than third metasomal tergite medially. Second suture finely crenulate. Third tergite almost entirely smooth but with traces of longitudinal striation near lateral parts of second suture. Thrid-fifth metasomal tergites distinctly arched in lateral profile. Ovipositor sheath 0.4 × length of hind tibia.

Colour. Stemmaticum and mesosoma entirely dark brown, nearly black; head, antennae (part remaining) and legs pale brown-yellow; metasomal tergites brown. Wings hyaline with pale grey-brown venation.

#### Etymology.

Named after the senior author’s favourite film actor Brad Pitt, whose poster adorned the wall of her laboratory during her doctoral studies.

#### Male.

Unknown.

#### Distribution.

South Africa.

#### Host.

Unknown.

**Figures 1–4.**
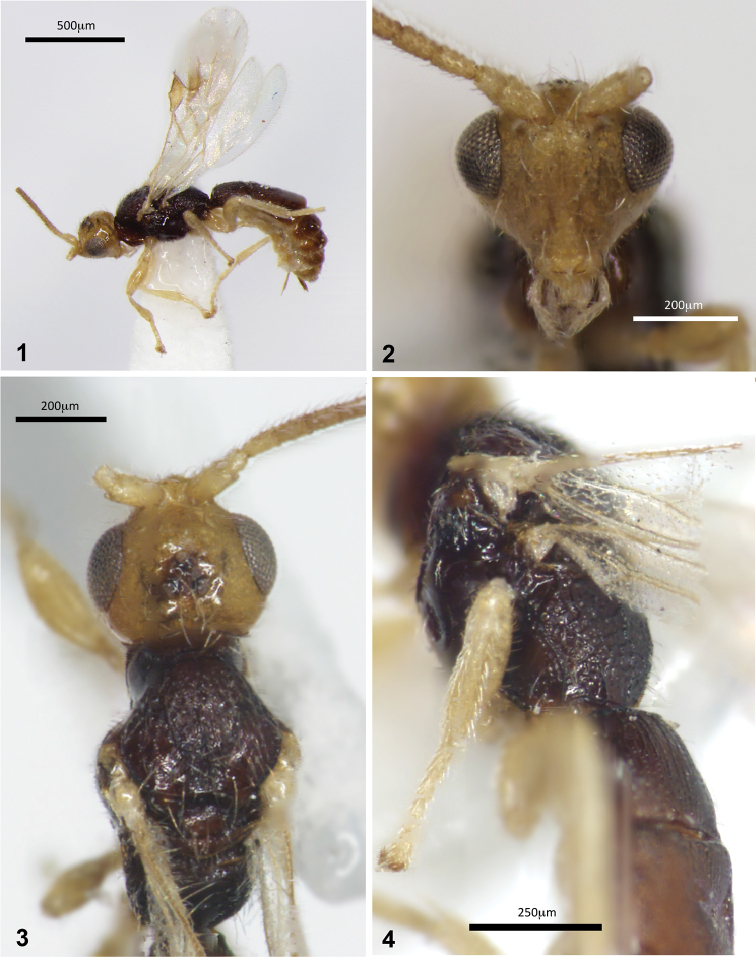
Montaged light micrographs of *Conobregma bradpitti* sp. n.; **1** habitus **2** face, anterior aspect **3** head and mesosoma, dorsal aspect **4** mesosoma, including propodeum, and anterior half of metasoma, oblique aspect.

**Figures 5–6.**
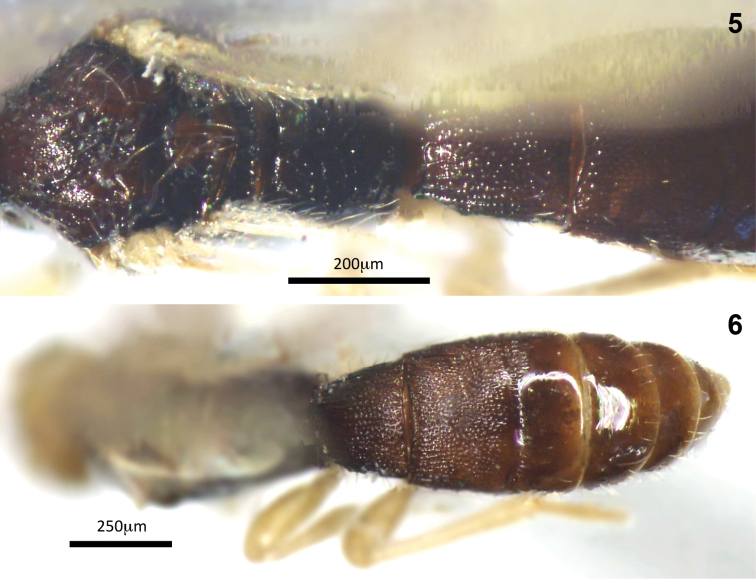
Montaged light micrographs of *Conobregma bradpitti* sp. n.; **5** mesosoma to tergite 2, dorsal aspect **6** metasoma, dorsal aspect.

### Facitorus nasseri

Taxon classificationAnimaliaHymenopteraBraconidae

Ranjith & Quicke sp. n.

http://zoobank.org/6739F6C6-62F4-44C4-B5C6-203F8B3895C3

Figures 7–12 , 13–15

#### Material examined.

Holotype, female, “India, Kerala, Malappuram, Calicut University Botanical Garden, 14–21.xii.2015, Malaise Trap, ex. Ranjith, A.P.”

#### Diagnosis.

*Facitorus nasseri* sp. n. is distinguished from *Facitorus brevicornis* van Achterberg and *Facitorus superus* van Achterberg in having occipital carina complete, mesoscutum covered by long setae and scutellum with sub-posterior depression. *Facitorus nasseri* sp. n. comes close to *Facitorus tamdaoensis* Belokobylskij & Long, by its smooth metasomal tergite 2, but it differs from *Facitorus tamdaoensis* by the following characters; mesoscutum sculptured antero-laterally (smooth in *Facitorus tamdaoensis*), frons without shallow pit medially (frons with shallow pit medially in *Facitorus tamdaoensis*), propodeum with ‘H’ shaped carina posteriorly (smooth in *Facitorus tamdaoensis*), pterostigma 2.9 × longer than maximum wide (3.6 × in *Facitorus tamdaoensis*) and second tergite entirely smooth (densely striate basally in *Facitorus tamdaoensis*). It differs from *Facitorus granulosus* and *Facitorus amamioshimus* by first flagellomere 2.1 × as long as apically wide (3.5–4.2 × in *Facitorus granulosus* and 3.5–4.0 × in *Facitorus amamioshimus*), second metasomal suture not crenulate (crenulate in *Facitorus granulosus* and *Facitorus amamioshimus*), third metasomal tergite entirely smooth (distinctly sculptured at least baso-laterally in *Facitorus granulosus* and *Facitorus amamioshimus*). A key for the identification of *Facitorus* species is presented below.

#### Description (female).

Holotype, female (♀), length of body 1.7 mm and fore wing 1.35 mm.

Head. Antennae with 18 segments. First flagellomere 1.2 × as long as second and third respectively, 2.1 × longer than apically wide, distinctly expanded from base to apex. Terminal flagellomere acute, 3.1 × as long as its maximum width. Width of head: width of face: height of eye = 13.4: 6.8: 7.1. Frons and occiput smooth with long setae. Eyes glabrous, with a straight groove between antennal socket and eye margin. Distance between posterior ocelli: transverse diameter of posterior ocellus: shortest distance between posterior ocellus and eye = 13.5: 10.25: 18.8. Occipital carina complete.

Mesosoma. Mesosoma 1.72 × longer than high. Propleuron smooth. Mesoscutum sculptured antero-laterally, smooth medio-posteriorly with long setae. Notauli impressed, meeting posteriorly and finely crenulate. Scutellar sulcus wide, deep and divided by a single carina. Scutellum smooth, sparsely setose with subposterior transverse depression. Median area of metanotum with medio-longitudinal ridge, rest smooth. Precoxal sulcus distinct only anteriorly impressed. Metapleuron medially smooth, rest rugose. Propodeum without medio-longitudinal carina, basal half distinctly foveate and with ‘H’ shaped carinae and transverse carinae. Pterostigma 2.9 × longer than maximally wide. Fore wing vein r-rs approximately 0.8 × maximum width of pterostigma. Lengths of fore wing veins r-rs: 3RSa: 3RSb = 2.8: 4.4: 12.5. Lengths of fore wing veins CU1a: CU1b = 3.25: 4.37. Fore femur: tibia: tarsus = 4.7: 4.58: 3.34. Fore basitarsus 1.6 × longer than next three articles combined. Mid-tibial claw well-developed, pectinate. Hind femur: tibia: tarsus = 5.4: 7.7: 7.0.

Metasoma. Metasomal tergite 1 distinctly striate, smooth medio-posteriorly, striae reaching posterior margin laterally, slightly convex apically, sparsely setose. Tergite 2 smooth, sparsely setose medially, setose laterally, 1.6 × as long as third tergite. Second metasomal suture slightly impressed, not crenulate. Tergite 3 smooth with a pair of setae medio-basally and postero-laterally. Rest of the tergite smooth, exposed in lateral view and sparsely setose. Ovipositor sheath setose and 0.42 × as long as hind tibia.

Colour. Body dark brown except scape, pedicel, first flagellomere, basal half of second flagellomere, maxillary palp, tegulae, legs and ovipositor yellow; face yellowish brown anteriorly below antennal sockets; propleuron ventrally yellowish brown; wings hyaline; pterostigma and venation light brown.

#### Etymology.

APR dedicates this species to Dr. M. Nasser for his encouragement and sharing his knowledge about the behaviour of parasitoids, and also for the fruitful discussions during the field trips.

#### Male.

Unknown.

#### Distribution.

India (Kerala).

#### Host.

Unknown.

**Figures 7–12.**
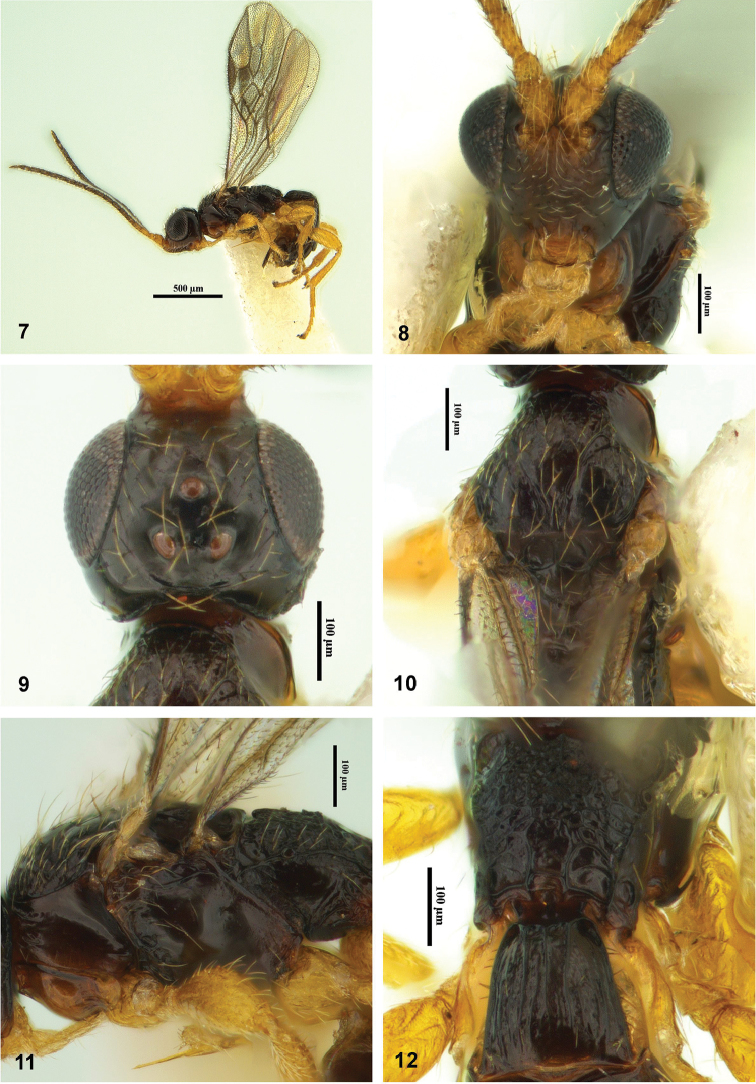
Montaged light micrographs of *Facitorus nasseri* sp. n.; **7** habitus **8** head, anterior aspect **9** head, dorsal aspecct **10** mesosoma, dorsal aspect **11** mesosoma, lateral aspect **12** propodeum and first metasomal tergite, dorsal aspect.

**Figures 13–15.**
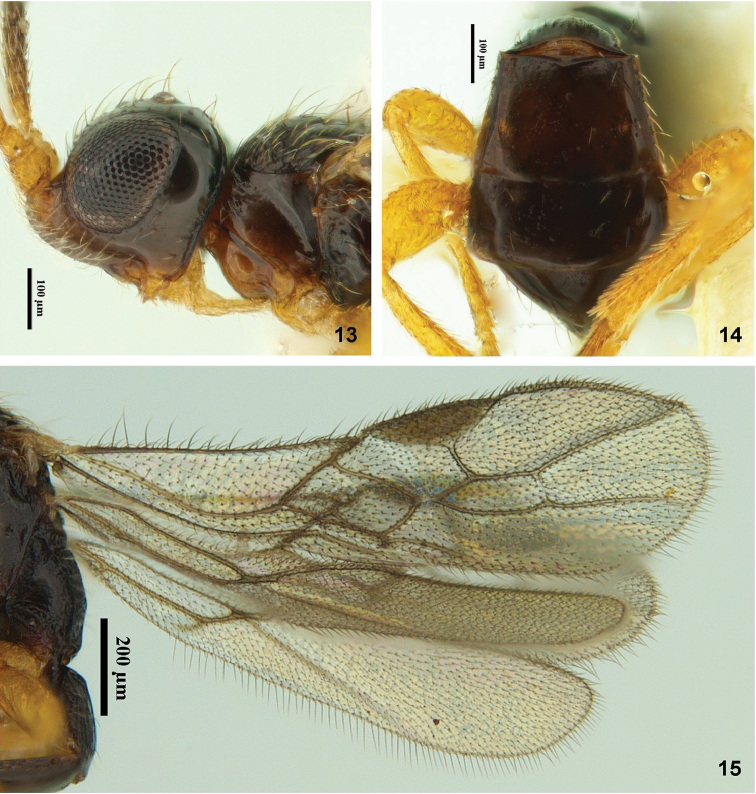
Montaged light micrographs of *Facitorus nasseri* sp. n.; **13** head & mesosoma (in part), lateral aspect **14** metasomal tergite 2 and following tergites, dorsal aspect **15** wings.

### Key to species of *Facitorus* van Achterberg

**Table:** 

1	Scutellum without sub-posterior depression; occipital carina interrupted medially; mesoscutum without long setae	**2**
–	Scutellum with subposterior depression; occipital carina complete; mesoscutum often covered by long setae	**3**
2	Fore wing vein r 1.5 × as long as 2-SR; malar space 2.8 × basal width of mandible; face sparsely punctate; second tergite largely smooth	***Facitorus brevicornis* van Achterberg**
–	Fore wing vein r almost equal to or shorter than 2-SR; malar space 2 × basal width of mandible; face smooth; second tergite rugose-punctate	***Facitorus superus* van Achterberg**
3	Mesoscutum entirely smooth or rugose antero-laterally; third tergite entirely smooth	**4**
–	Mesoscutum granulate; third tergite distinctly sculptured, at least baso-laterally	**5**
4	Mesoscutum rugose antero-laterally; transverse diameter of eye twice as long as temple; frons without shallow pit near antennal sockets; anterior half of propodeum foveate, with ‘H’ shaped carina posteriorly and transverse carina; pterostigma 2.9 × as long as its maximum width; hind coxa entirely smooth	***Facitorus nasseri* Ranjith & Quicke, sp. n.**
–	Mesoscutum entirely smooth; transverse diameter of eye 2.7 × as long as temple; frons with shallow pit near antennal sockets; propodeum densely rugose-reticulate; pterostigma 3.6 × as long as its maximum width; hind coxa rugose-striate laterally	***Facitorus tamdaoensis* Belokobylskij & Long**
5	Hind coxa dorsally striate; second metasomal suture deep; pterostigma enlarged, 1.1–1.2 × as long as R1; third tergite distinctly and widely sculptured; mesoscutum distinctly granulate	***Facitorus granulosus* Belokobylskij & Long**
–	Hind coxa entirely smooth; second metasomal suture shallow; pterostigma not enlarged, 0. 9× as long as R1; third tergite only baso-laterally finely striate or rugulose-strate; mesoscutum finely granulate	***Facitorus amamioshimus* Belokobylskij, Zaldivar-Riverón & Maetô**

## Discussion

*Conobregma bradpitti* sp. n. is the first record of the Facitorina from the African continent, the others occurring in the East Palaearctic, East Asia and North America (including Caribbean). The new species keys out easily to *Conobregma* in the generic key to Betylobraconi (as –inae) by van Achterberg (1985), but its characters are intermediate between those of *Conobregma* and the more recently described genus *Asiabregma* established by Belokobylskij et al. (2008). Originating from an intermediate location longitudinally, it may be not surprising that the new species displays a mix of character states between *Conobregma* and *Asiabregma* (Table 1). Differences between *Conobregma* and *Asiabregma* are in any case rather slight and probably would not normally be used to justify separate generic status had they not shown a disjunct distribution. With the discovery of the new species which shares two derived states with each nominal genus, we have to choose whether to arbitrarily assign it to one of them whilst keeping both separate though with reduced differences, creating a new genus for it based only on two small differences, or synonymising them. We have chosen the latter route because of the minimal differences, and therefore, we hereby formally synonymise *Asiabregma* Belokobylskij, 2008, with *Conobregma* van Achterberg, 1995.

*Facitorus nasseri* sp. n. is the first facitorine recorded from Indian subcontinent. The yeliconine subtribe Facitorina consists of the genera *Facitorus*, *Conobregma* and *Jannya* and they share the following characters; antennal sockets closer to each other than to eyes, frons without groove, antenna situated on a shelf, fore wing vein M+CU strongly curved apically (van Achterberg 1995a). *Facitorus* differs from the rest in having fore wing vein CU1a arising distinctly below the level of 2-CU1 and with comparatively large dorsope, but it shares a plesiomorphic character with *Conobregma* and *Jannya* of having a subposterior depression at the scutellum (Belokobylskij and Long 2005; Belokobylskij et al. 2008). All *Facitorus* species are distributed in the Oriental and South Palearctic Regions. The new species, *Facitorus nasseri* is different from its closest relative *Facitorus superus* (known from Nepal) in having the scutellum with the sub-posterior depression.

## Supplementary Material

XML Treatment for Conobregma bradpitti

XML Treatment for Facitorus nasseri
